# Integrated Metabolomic and Transcriptomic Analyses of Longissimus Thoracis Muscle Provide Insights into Metabolic Differences Between Yanbian and Yanhuang Cattle

**DOI:** 10.3390/biology15161356

**Published:** 2026-08-10

**Authors:** Yang Lyu, Zhiwei Zhu, Baoxin Zhao, Zezhu Ren, Meng Zhou, Jing Yu, He Ding, Hongyu Liu, Yi Fang, Jing Zhao, Wenfa Lyu

**Affiliations:** 1 Key Laboratory of the Animal Production, Product Quality and Security, Ministry of Education, Jilin Agricultural University, Changchun 130118, China; 15134378707@163.com (Y.L.); blue_we@126.com (Z.Z.); 18831040615@163.com (B.Z.); 18643801841@163.com (Z.R.); 15590756216@163.com (M.Z.); yuj19970614@163.com (J.Y.); dinghe1130@126.com (H.D.); jlndlhy0133@163.com (H.L.); 17743112414@163.com (Y.F.); 2 Jilin Provincial Key Laboratory of Beef Cattle Germplasm Resources Conservation and Utilization, Jilin Agricultural University, Changchun 130118, China; 3 Jilin Provincial International Joint Research Center of Animal Breeding and Reproduction Technology, Jilin Agricultural University, Changchun 130118, China

**Keywords:** cattle, metabolomics, transcriptomics, muscle metabolism, gene expression, meat quality

## Abstract

**Simple Summary:**

Meat quality is an important economic trait in beef cattle and is determined by complex interactions among genetic background, muscle metabolism, and post-mortem biochemical processes. Yanbian cattle, an indigenous breed from northeastern China, are known for desirable meat quality, whereas Yanhuang cattle were developed through crossbreeding to improve growth performance and carcass traits while retaining part of the Yanbian cattle genetic background. However, the molecular basis underlying the differences between these two cattle populations remains unclear. In this study, longissimus thoracis muscle samples from Yanbian and Yanhuang cattle were analyzed using integrated metabolomic, volatile compound, and transcriptomic approaches. Differences were detected in metabolites, volatile compounds, and gene expression profiles between the two breeds. Integrative analyses identified distinct gene–metabolite association patterns involving amino acid metabolism, carbohydrate metabolism, and lipid metabolism. These results provide candidate genes and metabolites for future studies aimed at understanding the molecular basis of meat quality variation in beef cattle.

**Abstract:**

Meat quality is an important economic trait in beef cattle and is influenced by breed-related metabolic characteristics. Yanbian cattle (YB) are valued for desirable meat quality, whereas Yanhuang cattle (YH), developed using Limousin cattle as the paternal line and Yanbian cattle as the maternal line, exhibit improved growth performance and carcass yield. However, the molecular basis underlying metabolic variation between these two genetically related cattle populations remains unclear. In this study, longissimus thoracis muscle samples from six animals per breed were analyzed using LC-MS/MS-based metabolomics and GC×GC-TOF/MS-based volatile compound profiling, and a subset of three samples per breed from the same cohort was selected for transcriptomic sequencing. A total of 1697 metabolites were detected. Based on the screening criteria of VIP > 1 and *p* < 0.05, 202 candidate metabolites were identified, among which 11 remained statistically significant after false discovery rate (FDR) correction. Volatile compound profiling detected 1333 and 1516 compounds in YB and YH, respectively, of which 809 were shared. Based on the same screening criteria, 39 candidate volatile compounds were identified, although none remained significant after FDR correction. Transcriptomic analysis identified 360 differentially expressed genes using |log_2_FC| > 1 and adjusted *p* < 0.05, with enrichment mainly observed in pathways associated with carbohydrate metabolism, lipid turnover, and energy utilization. Integrative analyses indicated that amino acid-related metabolites, including phenylpyruvate, 2-aminobenzoic acid, and asparagine, were more closely associated with candidate volatile compounds than metabolites involved in central carbon metabolism. Several genes involved in lipid metabolism, energy metabolism, and muscle structure, including *DGAT2*, *MGLL*, *CRYAB*, *CSRP3*, *AGPAT2*, *PHKA1*, *AMPD1*, and *PGM2L1*, were associated with distinct metabolic modules. These findings provide an exploratory view of breed-related metabolic variation and identify candidate molecular features for future validation.

## 1. Introduction

Meat quality is an important economic trait in beef cattle and is influenced by genetic background, muscle composition, metabolic activity, and post-mortem biochemical changes [1]. Differences in meat quality among cattle breeds are often associated with variation in muscle metabolism, fat deposition, and the accumulation of metabolites related to muscle biochemical characteristics [2,3,4]. In particular, amino acid metabolism, lipid turnover, and energy metabolism are closely linked to metabolic processes that influence muscle composition and the formation of volatile compounds during thermal processing [5,6]. These metabolic processes are regulated by gene expression programs involved in carbohydrate metabolism, amino acid turnover, lipid metabolism, and cellular energy utilization [7].

Recent advances in high-throughput omics technologies have provided new opportunities for investigating the molecular basis of complex livestock traits [8]. Metabolomics enables the characterization of small molecules associated with metabolic activity [9], whereas transcriptomics provides insight into gene expression patterns underlying physiological and metabolic variation [10]. Volatile compound profiling further facilitates the assessment of compounds generated through muscle biochemical reactions during thermal treatment [11]. Integrating multiple omics datasets enables coordinated molecular changes to be examined across different biological levels and provides a more comprehensive understanding of the molecular basis underlying economically important traits in livestock [12,13].

China possesses abundant indigenous cattle genetic resources with diverse production and meat quality characteristics [14,15]. Yanbian cattle (YB), a representative indigenous breed from northeastern China, are known for desirable meat quality and good adaptation to regional production conditions [16]. Yanhuang cattle (YH) were developed through crossbreeding using Limousin cattle as the paternal line and Yanbian cattle as the maternal line, followed by long-term population stabilization and selective breeding [17]. Compared with YB, YH exhibit improved growth performance and carcass yield [17]. Although the two breeds share part of their genetic background, differences in growth performance and muscle metabolism suggest that they may also differ in their underlying molecular characteristics. However, the molecular basis underlying these breed-related differences remains unclear, particularly regarding the coordinated variation in metabolites, volatile compounds, and gene expression. Therefore, an integrated multi-omics approach provides an opportunity to investigate coordinated molecular variation across multiple biological levels.

In the present study, integrated metabolomic, volatile compound, and transcriptomic analyses were performed to compare molecular characteristics in the longissimus thoracis muscle of YB and YH cattle. The objective was to identify candidate metabolites, candidate volatile compounds, and gene expression patterns associated with breed-related metabolic variation, and to characterize the molecular features underlying differences between the two breeds.

## 2. Materials and Methods

### 2.1. Animals and Sample Collection

Twelve healthy 24-month-old steers were included in this study, comprising six Yanbian cattle (YB1–YB6; average live weight: 491.75 ± 30.22 kg) and six Yanhuang cattle (YH1–YH6; average live weight: 575.64 ± 41.81 kg). All animals were born and raised on the same farm in Yanbian, Jilin Province, China, under identical feeding and management conditions from birth until slaughter, with free access to water. Animals were slaughtered according to the Chinese national standard for cattle processing (GB/T 19477–2018) [18]. Immediately after carcass splitting, longissimus thoracis (LT) muscle samples were collected between the 12th and 13th ribs, snap-frozen in liquid nitrogen, and stored at −80 °C until analysis. Samples from all twelve animals were used for metabolomic and volatile compound analyses, while three samples from each breed with high RNA quality and integrity were subsequently selected for transcriptomic sequencing. The experimental workflow is shown in Supplementary Figure S1. All animal procedures were approved by the Animal Ethics Committee of Jilin Agricultural University (Approval No. 20250317001) and conducted in accordance with relevant guidelines and regulations.

### 2.2. Metabolomics Analysis

Metabolomic profiling was performed using an LC-MS/MS platform (Thermo Fisher Scientific, Waltham, MA, USA) operating in both positive and negative ion modes. Sample preparation, metabolite extraction, chromatographic separation, and mass spectrometric conditions are described in Supplementary Methods S1. Raw data were converted using ProteoWizard (v3.0.8789) [19] and processed using the XCMS (v3.12.0) [20] package in R for peak detection, retention time correction, alignment, and normalization. Metabolite annotation was performed using accurate mass measurements and MS/MS spectra. Principal component analysis (PCA) and orthogonal partial least squares discriminant analysis (OPLS-DA) were conducted using the ropls package (v1.22.0) in R [21]. Model robustness was evaluated using 200 permutation tests. Candidate metabolites were identified using the combined criteria of VIP > 1 and *p* < 0.05. Benjamini–Hochberg false discovery rate (FDR) correction was further applied to evaluate statistical robustness. Candidate metabolites were subsequently used for exploratory pathway enrichment and integrative analyses. Pathway enrichment analysis was conducted using a hypergeometric test combined with pathway topology analysis [22].

### 2.3. Volatile Compound Profiling

Volatile compounds were analyzed using headspace solid-phase microextraction combined with comprehensive two-dimensional gas chromatography–time-of-flight mass spectrometry (GC×GC-TOF/MS, LECO Pegasus 4D). Detailed sample preparation, extraction conditions, chromatographic separation, and mass spectrometric parameters are described in Supplementary Methods S2. Compounds were identified using the NIST 2020 mass spectral library together with retention index matching based on C7–C30 n-alkanes. Compound classification was performed using PubChem and ClassyFire databases [23], and odor descriptors were assigned according to FlavorDB [24]. PCA and OPLS-DA were performed using SIMCA-P (v13.0) and the ropls package in R. Candidate volatile compounds were identified using VIP > 1 and *p* < 0.05, where *p* values were calculated using Student’s *t*-test. Benjamini–Hochberg FDR correction was subsequently applied. Candidate volatile compounds were used for exploratory characterization and integrative analyses.

### 2.4. Transcriptomic Sequencing and Analysis

Total RNA was extracted from LT muscle using TRIzol reagent (Invitrogen, Carlsbad, CA, USA), and RNA integrity was evaluated using an Agilent 2100 Bioanalyzer (Agilent Technologies, Santa Clara, CA, USA). RNA samples with high integrity were used for library preparation. RNA sequencing libraries were prepared using the NEBNext^®^ Ultra™ RNA Library Prep Kit (New England Biolabs, Ipswich, MA, USA) and sequenced on an Illumina NovaSeq 6000 platform (Illumina, San Diego, CA, USA) with paired-end 150 bp reads. Raw reads were quality-filtered using Cutadapt (v1.9.1) and aligned to the *Bos taurus* reference genome (ARS-UCD1.2) using HISAT2 (v2.2.1). Gene expression levels were quantified using HTSeq (v0.6.1). Differential expression analysis was performed using DESeq2 (v1.26.0) based on raw read counts. Differentially expressed genes (DEGs) were identified using |log_2_FC| > 1 and adjusted *p* < 0.05. GO and KEGG enrichment analyses were subsequently performed using KOBAS-i [25].

### 2.5. Multi-Omics Integration Analysis

Multi-omics integration was performed to investigate potential associations among DEGs, candidate metabolites, and candidate volatile compounds. Representative DEGs were selected from significantly enriched KEGG pathways based on differential expression significance, pathway enrichment, and biological relevance. Candidate metabolites were selected from exploratory metabolomic pathway analysis according to VIP > 1 and *p* < 0.05. Normalized gene expression values, metabolite abundances, and volatile compound abundances were used for Pearson correlation analysis. Pearson correlation coefficients with *p* < 0.05 were retained for exploratory visualization. Correlation heatmaps were generated using the pheatmap package (v1.0.12) in R (v4.3.2), and KEGG enrichment results were visualized using ggplot2 (v3.5.1).

### 2.6. Statistical Analysis

Student’s *t*-test was used to compare metabolite and volatile compound abundances between breeds. Candidate metabolites and volatile compounds were screened using the combined criteria of VIP > 1 and *p* < 0.05, followed by Benjamini–Hochberg FDR correction to evaluate statistical robustness. Features identified by the exploratory screening criteria were retained for pathway enrichment and multi-omics integration analyses.

## 3. Results

### 3.1. Metabolomic Profiling of Yanbian and Yanhuang Cattle

**Figure 1:**
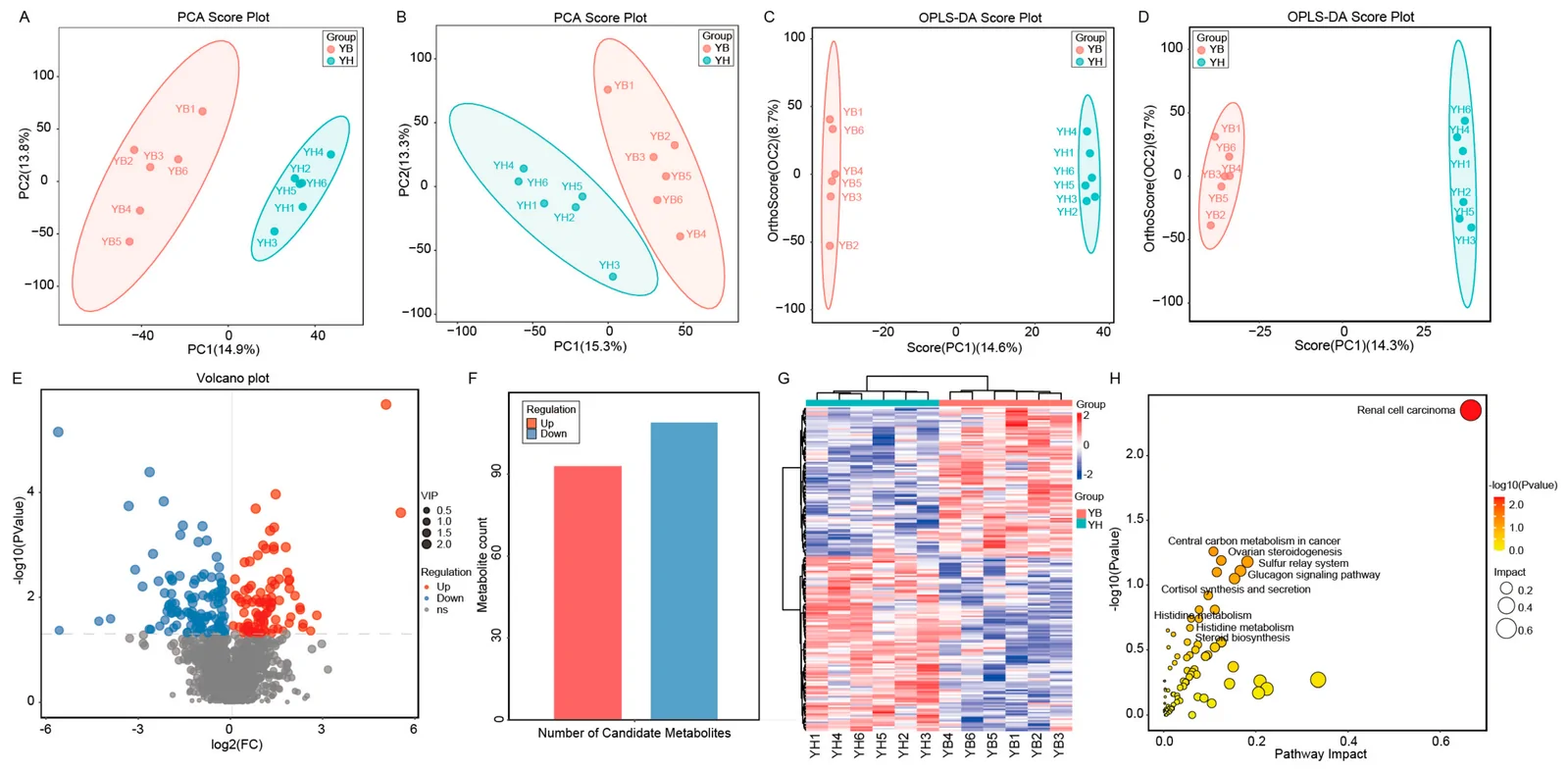
Metabolomic profiling of longissimus thoracis muscle in Yanbian (YB) and Yanhuang (YH) cattle. (**A**) PCA score plot in positive-ion mode. (**B**) PCA score plot in negative-ion mode. (**C**) OPLS-DA score plot in positive-ion mode. (**D**) OPLS-DA score plot in negative-ion mode. (**E**) Volcano plot of candidate metabolites identified using VIP > 1 and *p* < 0.05. Red and blue indicate metabolites with higher and lower abundance in YH relative to YB, respectively. (**F**) Numbers of candidate metabolites with higher and lower abundance in YH relative to YB. (**G**) Hierarchical clustering heatmap of the 202 candidate metabolites. (**H**) Exploratory KEGG pathway analysis of the 202 candidate metabolites identified using VIP > 1 and *p* < 0.05.

Untargeted metabolomics was performed to compare the metabolic profiles of LT muscle samples from six YB and six YH cattle. PCA score plots showed separation between the two groups in both the positive-ion mode (Figure 1A) and the negative-ion mode (Figure 1B), indicating overall differences in metabolite profiles of YB and YH cattle. OPLS-DA score plots also showed group separation in positive-ion mode (Figure 1C) and negative-ion mode (Figure 1D). Permutation tests further supported the stability of the OPLS-DA models (Figures S2 and S3).

A total of 1697 metabolites were detected using LC–MS/MS (Table S1). Based on the screening criteria of VIP > 1 and *p* < 0.05, 202 candidate metabolites were identified, including 93 with higher abundance and 109 with lower abundance in YH relative to YB (Figure 1E–G and Table S2). Among these candidate metabolites, 11 remained significant after Benjamini–Hochberg false discovery rate correction (FDR < 0.05). The complete set of 202 candidate metabolites was retained for exploratory pathway enrichment and downstream integrative analyses. The candidate metabolites included amino acids and their derivatives, lipids, organic acids, and carbohydrate-related metabolites. Exploratory KEGG analysis of the 202 candidate metabolites identified pathways related to steroid biosynthesis, arginine biosynthesis, histidine metabolism, and glucagon signaling (Figure 1H). Several phosphatidylcholines (PCs) and sphingomyelins (SMs) showed higher abundance in YH, whereas amino acid-related metabolites showed higher abundance in YB.

### 3.2. Volatile Compound Profiling of Yanbian and Yanhuang Cattle

**Figure 2:**
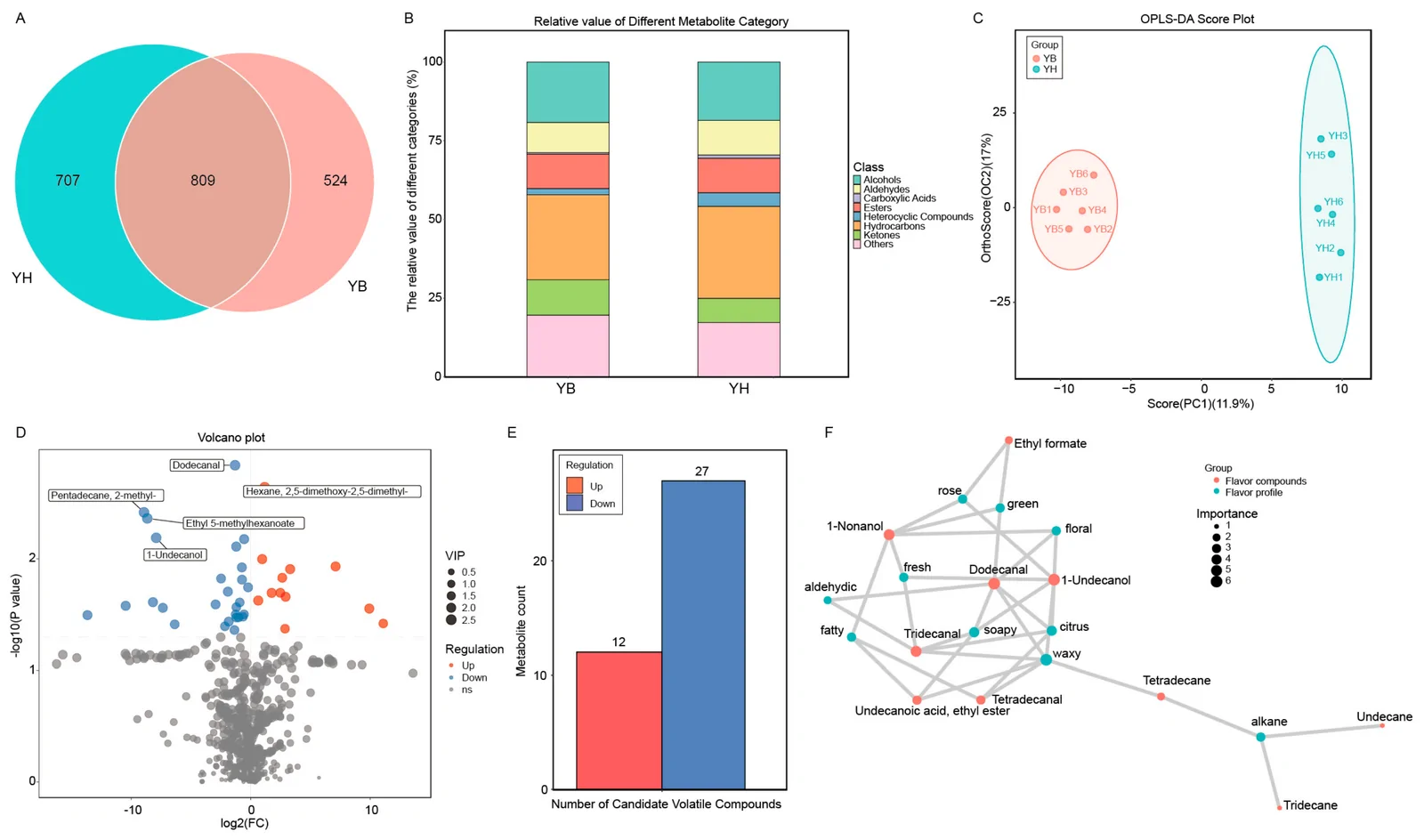
Volatile compound profiling of longissimus thoracis muscle in Yanbian (YB) and Yanhuang (YH) cattle. (**A**) Venn diagram showing volatile compounds detected in YB and YH and those shared between the two groups. (**B**) Classification and relative abundance of volatile compound categories. (**C**) OPLS-DA score plot of volatile compound profiles in YB and YH. (**D**) Volcano plot of candidate volatile compounds identified using VIP > 1 and *p* < 0.05. (**E**) Number of candidate volatile compounds with higher (Up) or lower (Down) abundance in YH relative to YB. (**F**) Network linking candidate volatile compounds with FlavorDB-derived odor descriptors.

GC×GC-TOF/MS analysis detected 1333 volatile compounds in YB and 1516 in YH, of which 809 were shared between the two groups (Figure 2A). The detected compounds were mainly classified as hydrocarbons, alcohols, aldehydes, ketones, esters, and heterocyclic compounds (Figure 2B). Hydrocarbons accounted for the largest proportion in both groups, while the relative abundances of aldehydes, alcohols, ketones, and heterocyclic compounds varied between YB and YH (Table S3). PCA showed partial separation between the two groups (Figure S4), and the OPLS-DA score plot showed separation of YB and YH samples based on their volatile compound profiles (Figure 2C). Using the screening criteria of VIP > 1 and *p* < 0.05, 39 candidate volatile compounds were identified, including 12 with higher abundance and 27 with lower abundance in YH relative to YB (Figure 2D,E and Table S4). None of these candidate volatile compounds remained significant after Benjamini–Hochberg false discovery rate correction. The candidate compounds mainly included aldehydes, alcohols, esters, and hydrocarbons. Their abundance patterns across samples are shown in the hierarchical clustering heatmap (Figure S5).

Representative candidate volatile compounds included dodecanal, tridecanal, tetradecanal, 13-methyltetradecanal, 1-nonanol, 1-undecanol, ethyl formate, and ethyl 5-methylhexanoate. FlavorDB annotation linked these compounds to odor descriptors including waxy, fatty, citrus, floral, and green (Figure S6). The relationships between the candidate volatile compounds and their annotated odor descriptors are summarized in the compound–odor descriptor network (Figure 2F).

### 3.3. Transcriptomic Profiling of Yanbian and Yanhuang Cattle

**Figure 3:**
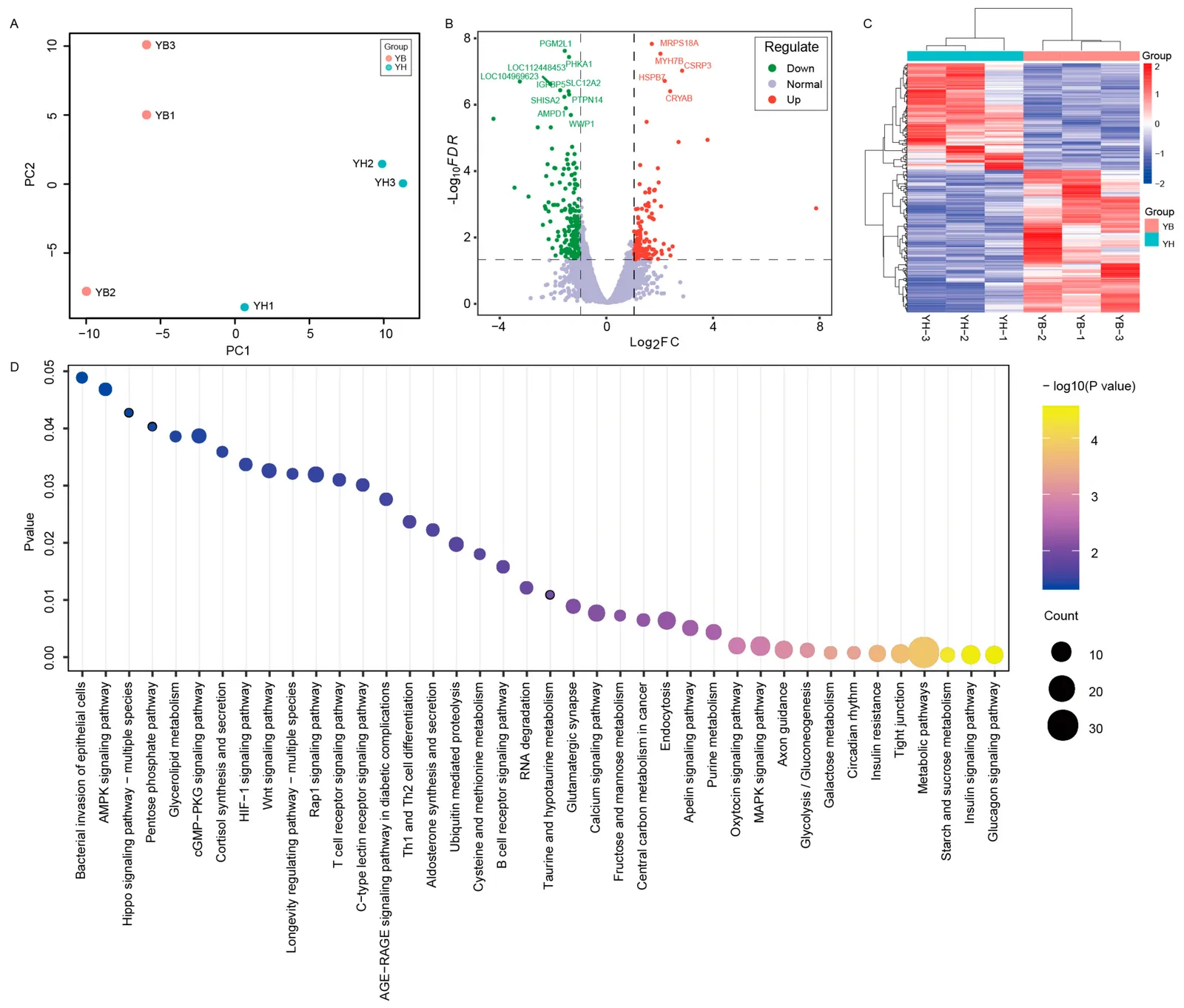
Transcriptomic profiling of longissimus thoracis muscle in Yanbian (YB) and Yanhuang (YH) cattle. (**A**) PCA plot based on normalized gene expression profiles, with colors indicating the YB and YH groups. (**B**) Volcano plot of differentially expressed genes (DEGs) identified using |log_2_FC| > 1 and adjusted *p* < 0.05. (**C**) Hierarchical clustering heatmap of DEGs. (**D**) KEGG pathway enrichment analysis of DEGs. Bubble size represents the number of genes enriched in each pathway, and color indicates the enrichment significance.

RNA sequencing was performed to investigate transcriptional differences between YB and YH cattle. PCA based on normalized gene expression profiles showed separation of the two groups along the first principal component (Figure 3A). Differential expression analysis identified 360 DEGs (|log_2_FC| > 1 and adjusted *p* < 0.05), including 169 upregulated and 191 downregulated genes in YH relative to YB (Figure 3B and Table S5). Representative DEGs included *MRPS18A*, *MYH7B*, *CRYAB*, *CSRP3*, *HSPB7*, *PHKA1*, *PGM2L1*, *AMPD1*, and *SLC12A2*. Hierarchical clustering of the DEGs showed clear separation between YB and YH samples based on their gene expression profiles (Figure 3C).

KEGG enrichment analysis showed that the DEGs were significantly enriched in pathways related to glycolysis/gluconeogenesis, glucagon signaling, insulin signaling, glycerolipid metabolism, pentose phosphate pathway, and AMPK signaling (Figure 3D and Table S6). GO enrichment analysis showed enrichment in biological processes associated with glycogen metabolism, lipid catabolism, glucose metabolism, and carbohydrate metabolism. The enriched cellular component terms included mitochondrion and cytosol, whereas ATP binding and protein kinase activity were among the most significantly enriched molecular functions (Table S7).

### 3.4. Integrated Analysis of Transcriptomic and Metabolomic Data

**Figure 4:**
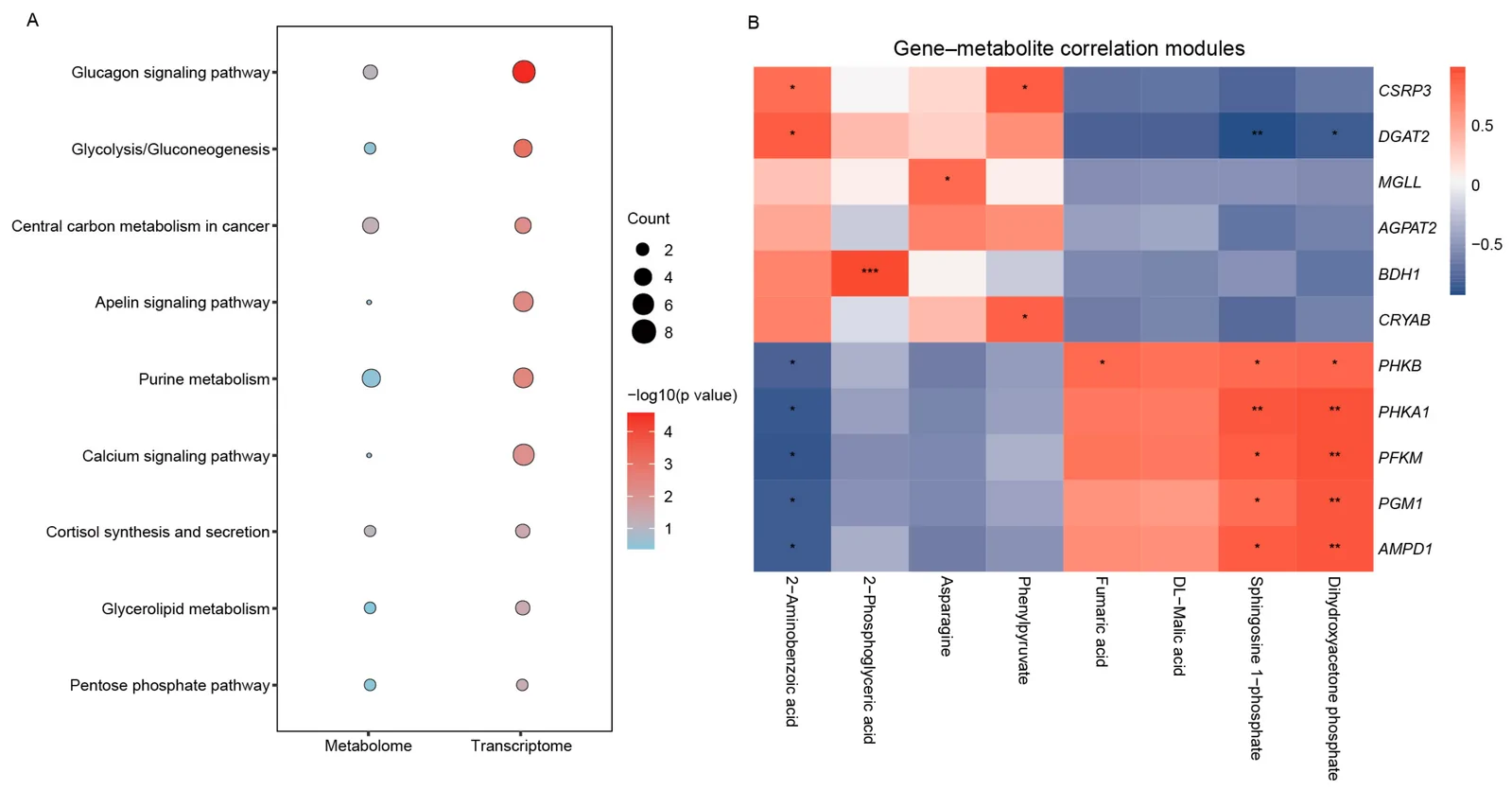
Integrated transcriptomic–metabolomic analysis of Yanbian (YB) and Yanhuang (YH) cattle. (**A**) Comparative KEGG pathway analysis of transcriptomic and metabolomic datasets. Bubble size represents the number of genes or metabolites, and color indicates −log10(*p* value). (**B**) Correlation heatmap showing associations between representative differentially expressed genes and selected candidate metabolites. Red and blue indicate positive and negative correlations, respectively. Asterisks indicate significance levels based on *p* values (* *p* < 0.05, ** *p* < 0.01, *** *p* < 0.001).

To investigate relationships between transcriptomic and metabolomic profiles, an integrative analysis was performed. Comparative KEGG pathway analysis indicated several pathways shared between the transcriptomic and metabolomic datasets, including glycolysis/gluconeogenesis, glucagon signaling, glycerolipid metabolism, the pentose phosphate pathway, and central carbon metabolism (Figure 4A). Pearson correlation analysis identified two major gene–metabolite correlation modules (Figure 4B). One module comprised genes involved in carbohydrate metabolism, including *PHKA1*, *PHKB*, *PFKM*, *PGM1*, and *AMPD1*, which showed positive correlations with fumaric acid, DL-malic acid, and dihydroxyacetone phosphate. In contrast, *DGAT2*, *MGLL*, *AGPAT2*, *BDH1*, *CRYAB*, and *CSRP3* formed a second module that showed positive correlations with amino acid-related metabolites, including phenylpyruvate, asparagine, and 2-aminobenzoic acid. Pearson correlation analysis between candidate metabolites and candidate volatile compounds is presented in Figure S7. Several amino acid-related candidate metabolites showed correlations with a larger number of candidate volatile compounds than did central carbon metabolites.

## 4. Discussion

Meat quality differences among cattle breeds are closely associated with variation in muscle metabolism and the accumulation of metabolites involved in post-mortem biochemical processes [4,26]. In the present study, integrated metabolomic and transcriptomic analyses suggested distinct molecular characteristics between Yanbian (YB) and Yanhuang (YH) cattle. Although the two breeds shared a large proportion of detected metabolites and volatile compounds, differences were observed in candidate metabolites, gene expression profiles, and several metabolic pathways. Pathways related to amino acid metabolism, carbohydrate metabolism, and lipid metabolism, including arginine biosynthesis, histidine metabolism, glycolysis/gluconeogenesis, glucagon signaling, and glycerolipid metabolism, were consistently identified in both metabolomic and transcriptomic analyses. These observations indicate coordinated changes in metabolite abundance and gene expression between the two breeds, although additional validation is required to determine their biological significance.

Metabolomic analysis identified several candidate metabolites that were enriched in pathways associated with amino acid metabolism, carbohydrate metabolism, and metabolic regulation. Among these metabolites, phenylpyruvate, asparagine, and 2-aminobenzoic acid were associated with amino acid metabolism, whereas fumaric acid, malic acid, and dihydroxyacetone phosphate represented intermediates of central carbon metabolism. Amino acid metabolism is an important component of muscle biochemical processes because amino acid-derived intermediates participate in multiple metabolic reactions and contribute to protein turnover and cellular metabolism [27,28]. Phenylpyruvate, an intermediate of phenylalanine metabolism, has been associated with amino acid catabolism and metabolic activity in skeletal muscle, whereas asparagine participates in amino acid biosynthesis and nitrogen metabolism [29,30,31]. In contrast, fumaric acid, malic acid, and dihydroxyacetone phosphate are key intermediates of central carbon metabolism and are generally regarded as indicators of cellular energy status and metabolic activity [32,33]. Together, these observations indicate that candidate metabolites associated with amino acid metabolism and central carbon metabolism differed between the two breeds.

Transcriptomic analysis provided complementary evidence for the observed metabolic patterns. Genes involved in glycolysis and glycogen metabolism, including *PHKA1* [34], *PHKB* [35], *PFKM* [36], and *PGM1* [37], showed positive correlations with fumaric acid, malic acid, and dihydroxyacetone phosphate, forming a carbohydrate metabolism-related module. These genes participate in glycogen utilization and glucose metabolism and are closely linked to pathways such as glycolysis/gluconeogenesis and glucagon signaling. In comparison, genes associated with lipid metabolism, energy utilization, stress response, and muscle structure, including *DGAT2* [38], *MGLL* [39], *AGPAT2* [40], *BDH1* [41], *CRYAB* [42], and *CSRP3* [43], were linked to amino acid-related metabolites such as phenylpyruvate, asparagine, and 2-aminobenzoic acid. Because the metabolomic and volatile compound analyses were exploratory and the transcriptomic analysis included three biological replicates per breed, these correlations should be considered hypothesis-generating and require validation in larger populations and functional studies. These correlation patterns are consistent with differences in carbohydrate, lipid, and amino acid metabolism between the two breeds.

The comparison between YB and YH also provides insight from a breeding perspective. Yanhuang cattle were developed using Limousin cattle as the paternal line and Yanbian cattle as the maternal line, followed by long-term population stabilization and selective breeding [17]. Although YH shows improved growth performance and carcass traits, it still retains part of the Yanbian cattle genetic background. Consistent with this breeding history, many metabolites and enriched pathways were shared between the two breeds, whereas differences were observed in specific metabolites, gene expression patterns, and gene–metabolite associations. These observations suggest that selective breeding may influence particular metabolic processes while preserving many molecular characteristics inherited from the founder population. Because Limousin cattle were not included in the present study, the observed molecular differences cannot be directly attributed to Limousin introgression alone. Instead, they are more likely to reflect the combined effects of breed composition and long-term artificial selection during the development of Yanhuang cattle.

Although the present study focused on molecular characteristics rather than production performance, the candidate metabolites, genes, and pathways identified here provide a molecular resource for future investigations of beef quality. Following validation in larger populations and integration with production and sensory traits, these candidate molecular features may facilitate biomarker discovery and support future molecular-assisted breeding strategies for beef quality improvement.

## 5. Conclusions

Integrated metabolomic, volatile compound, and transcriptomic analyses suggested molecular differences between Yanbian and Yanhuang cattle. Comparative multi-omics analysis identified candidate metabolites, genes, and metabolic pathways associated with amino acid, carbohydrate, and lipid metabolism, together with distinct gene–metabolite correlation patterns between the two breeds. These findings provide new molecular insights into breed-related metabolic variation and establish a foundation for future studies on the molecular basis of beef quality traits.

## Data Availability

The raw data supporting the conclusions of this article will be made available by the authors on request.

## Supplementary Materials

The following supporting information can be downloaded at: https://www.mdpi.com/article/10.3390/biology15161356/s1, Method S1: LC–MS/MS metabolomic analysis; Method S2: HS-SPME–GC×GC-TOF/MS analysis; Figure S1: Overview of the experimental design and multi-omics analytical workflow; Figure S2: Permutation test of the OPLS-DA model in negative ion mode; Figure S3: Permutation test of the OPLS-DA model in positive ion mode; Figure S4: PCA score plot of volatile compounds in Yanbian (YB) and Yanhuang (YH) cattle; Figure S5: Hierarchical clustering heatmap of candidate volatile compounds; Figure S6. FlavorDB-derived odor descriptor profiles of candidate volatile compounds between Yanbian (YB) and Yanhuang (YH) cattle; Figure S7: Correlation analysis between candidate metabolites and candidate volatile compounds; Table S1: Complete list of identified metabolites detected by LC-MS/MS; Table S2: Candidate metabolites between Yanbian (YB) and Yanhuang (YH) cattle; Table S3: Classification and relative abundance of volatile compounds; Table S4: Candidate volatile compounds identified using VIP > 1 and *p* < 0.05; Table S5: List of differentially expressed genes between YB and YH; Table S6: KEGG pathway enrichment analysis of DEGs; Table S7: GO terms enrichment analysis of DEGs.

## Abbreviations

The following abbreviations are used in this manuscript:

DEGs	Differentially expressed genes
FC	Fold change
GO	Gene Ontology
LT	longissimus thoracis
OPLS-DA	Orthogonal partial least squares discriminant analysis
PCA	Principal component analysis
YB	Yanbian cattle
YH	Yanhuang cattle
