# Supplementary material for: Integrated Metabolomic and Transcriptomic Analyses of Longissimus Thoracis Muscle Provide Insights into Metabolic Differences Between Yanbian and Yanhuang Cattle
Source: Biology (Basel). 2026 Aug 10;15(16):1356. doi: 10.3390/biology15161356 (PMC13509357; doi:10.3390/biology15161356)
Supplement: Supplementary file 1 [file biology-15-01356-s001.zip › Supplementary Materials.pdf]

Supplementary Materials

Supplementary Methods:

Method S1. LC－MS/MS metabolomic analysis

Method S2. HS-SPME–GC×GC-TOF/MS analysis

Supplementary Tables:

Table S1. Complete list of identified metabolites detected by LC-MS/MS.

Table S2. Candidate metabolites between Yanbian (YB) and Yanhuang (YH) cattle.

Table S3. Classification and relative abundance of volatile compounds.

Table S4. Candidate volatile compounds identified using VIP > 1 and $P < 0.05$.

Table S5. List of differentially expressed genes between YB and YH.

Table S6. KEGG pathway enrichment analysis of DEGs.

Table S7. GO terms enrichment analysis of DEGs.

Supplementary Figures:

Figure S1. Overview of the experimental design and multi-omics analytical workflow.

Figure S2. Permutation test of the OPLS-DA model in negative ion mode.

Figure S3. Permutation test of the OPLS-DA model in positive ion mode.

Figure S4. PCA score plot of volatile compounds in Yanbian (YB) and Yanhuang (YH) cattle.

Figure S5. Hierarchical clustering heatmap of candidate volatile compounds.

Figure S6. FlavorDB-derived odor descriptor profiles of candidate volatile compounds between Yanbian (YB) and Yanhuang (YH) cattle.

Figure S7. Correlation analysis between candidate metabolites and candidate volatile compounds.

Supplementary Methods

Method S1. LC–MS/MS metabolomic analysis

Metabolites were extracted from 50 mg of frozen longissimus thoracis (LT) muscle using 1 mL of methanol/chloroform/water (7.5:1:2.5, v/v/v). Following homogenization, sonication, and centrifugation (12,000 × g, 10 min, 4 °C), the supernatant was collected, vacuum-dried, and reconstituted in 100 μL of 50% acetonitrile containing 2-chloro-L-phenylalanine as an internal standard. Metabolomic profiling was performed using a Vanquish UHPLC system coupled with an Orbitrap Exploris 120 mass spectrometer (Thermo Fisher Scientific, Bremen, Germany). Chromatographic separation was achieved on an ACQUITY UPLC HSS T3 column (2.1 × 100 mm, 1.8 μm; Waters, Milford, MA, USA). The column temperature was maintained at 40 °C with a flow rate of 0.30 mL/min and an injection volume of 2 μL. Data were acquired in both positive- and negative-ion modes over an m/z range of 100–1000. Raw LC–MS data were converted into mzXML format using ProteoWizard (v3.0.8789). Peak detection, retention time correction, alignment, and peak integration were performed using the XCMS package (v3.12.0) with the centWave algorithm. Peak intensities were normalized using total peak area normalization before statistical analysis. Metabolites were annotated based on accurate mass measurements together with MS/MS spectra by comparison with the reference metabolite database.

Method S2. HS-SPME–GC×GC-TOF/MS analysis

Volatile compounds were analyzed using headspace solid-phase microextraction coupled with comprehensive two-dimensional gas chromatography–time-of-flight mass spectrometry (HS-SPME–GC×GC-TOF/MS). Approximately 0.5 g of minced LT muscle was transferred into a 20 mL headspace vial and mixed with 10 μL of n-hexanol-d13 internal standard solution (1 mg/mL in 50% ethanol). Samples were equilibrated at 80 °C for 10 min and extracted using a 50/30 μm DVB/CAR/PDMS SPME fiber (Supelco, Bellefonte, PA, USA) at 80 °C for 25 min. Volatile compounds were thermally desorbed in the GC injector at 250 °C for 5 min. GC×GC-TOF/MS analysis was performed using an Agilent 8890A gas chromatograph coupled with a LECO Pegasus BT 4D time-of-flight mass spectrometer (LECO, St. Joseph, MI, USA). A DB-Heavy Wax column was used as the first-dimension column, and an Rxi-5Sil MS column was used as the second-dimension column. Helium was used as the carrier gas at a constant flow rate of 1.0 mL/min. Mass spectra were acquired under electron ionization (70 eV) over an m/z range of 35–550. Raw chromatographic data were processed using ChromaTOF software. Compound identification was performed by comparison with the NIST 2020 mass spectral library together with retention index matching based on C7–C30 n-alkanes. Peak areas were normalized using the internal standard before statistical analysis. Compound classification and odor annotation were performed using the PubChem, ClassyFire, and FlavorDB databases.

Supplementary Figures

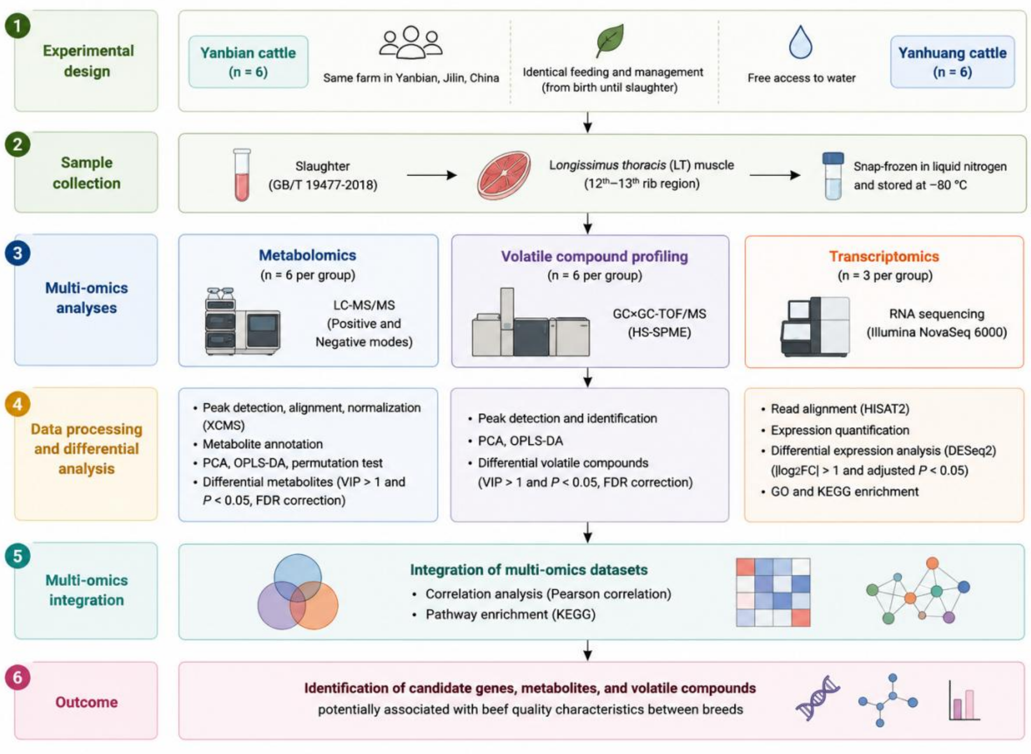

Figure S1. Overview of the experimental design and multi-omics analytical workflow.

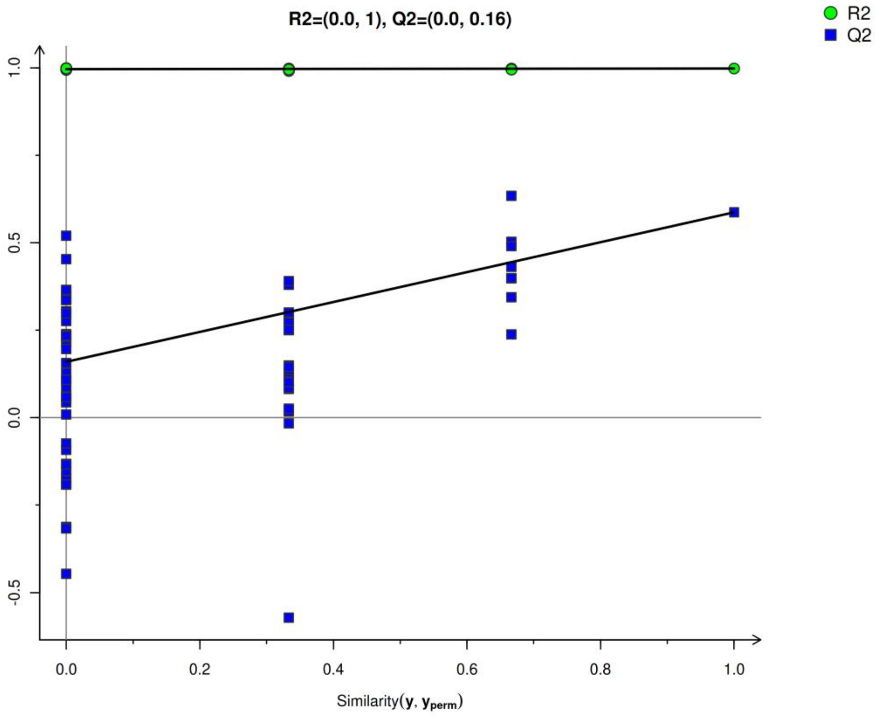

Figure S2. Permutation test of the OPLS-DA model in negative ion mode.

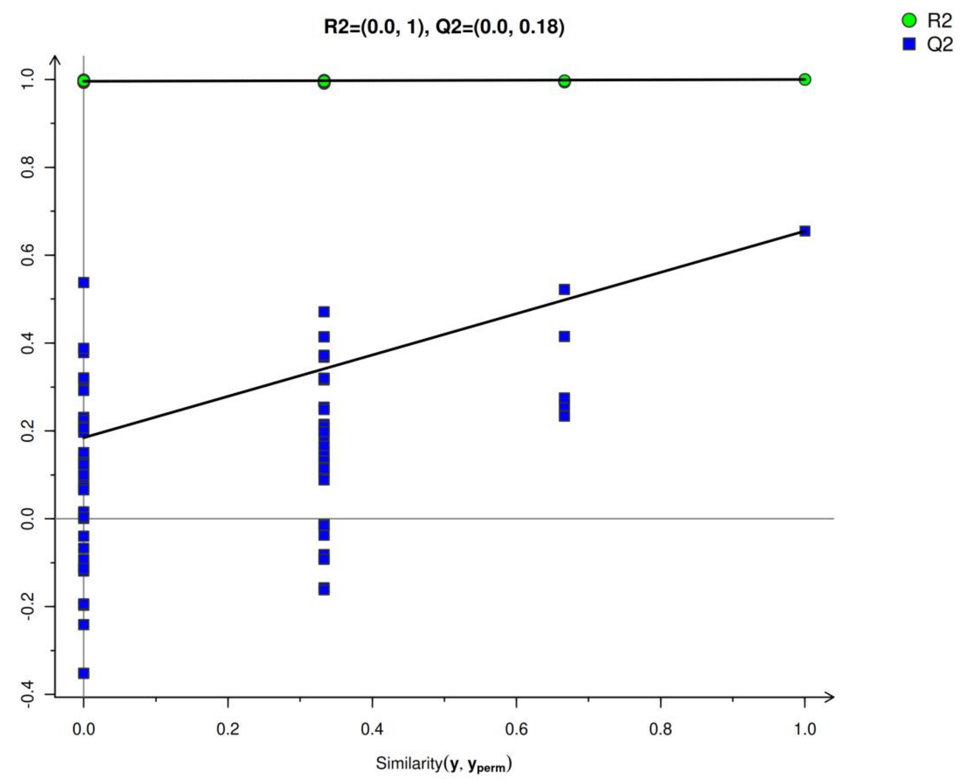

Figure S3. Permutation test of the OPLS-DA model in positive ion mode.

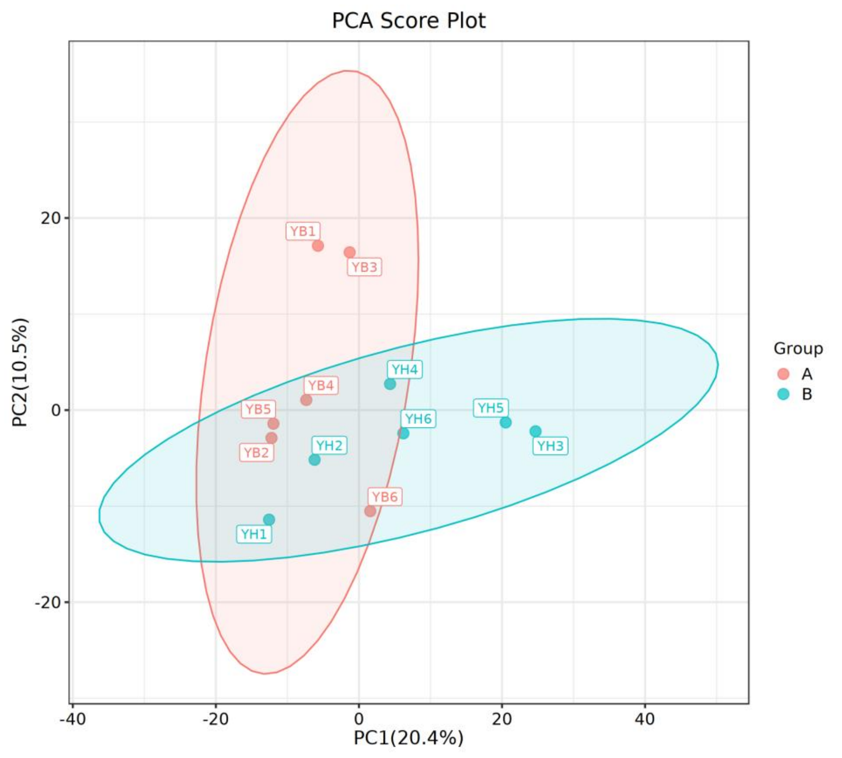

Figure S4. PCA score plot of volatile compounds in Yanbian (YB; A) and Yanhuang (YH; B) cattle.

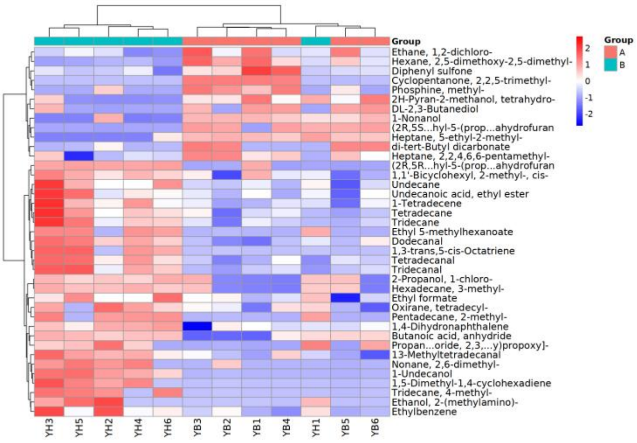

Figure S5. Hierarchical clustering heatmap of candidate volatile compounds.

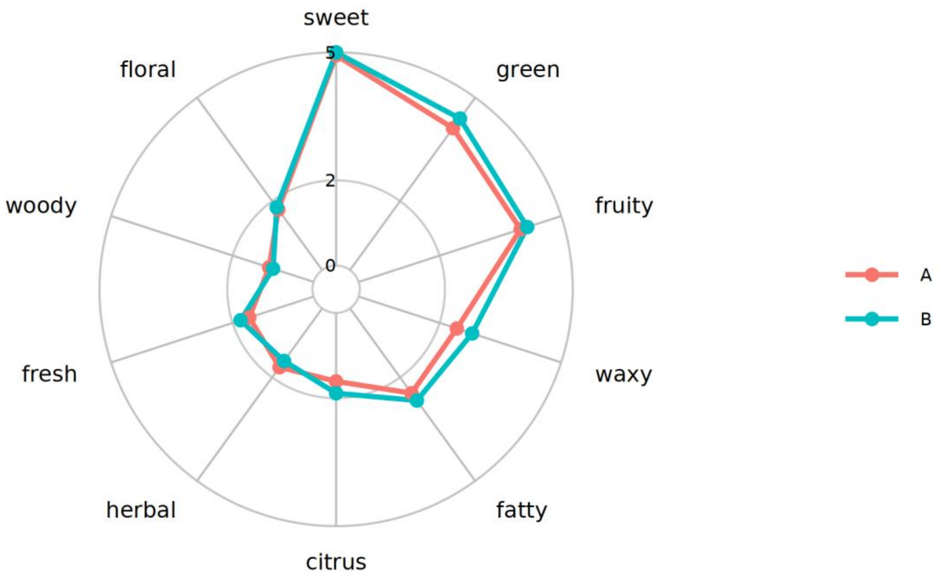

Figure S6. FlavorDB-derived odor descriptor profiles of candidate volatile compounds between Yanbian (YB; A) and Yanhuang (YH; B) cattle.

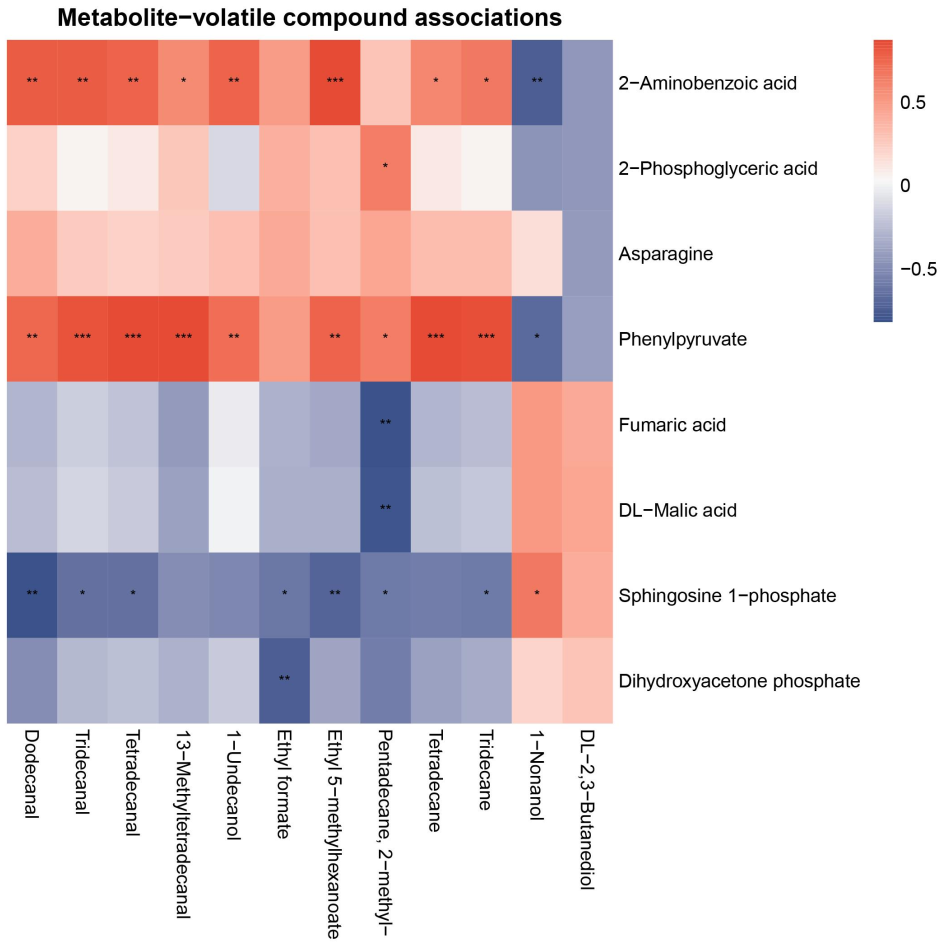

Figure S7. Correlation analysis between candidate metabolites and candidate volatile compounds.
